# Effectiveness of three modes of kinetic-chain exercises on quadriceps muscle strength and thigh girth among individuals with knee osteoarthritis

**DOI:** 10.1186/s40945-017-0036-6

**Published:** 2017-07-19

**Authors:** Oladapo Michael Olagbegi, Babatunde Olusola Adegoke, Adesola Christiana Odole

**Affiliations:** 1 Department of Human Kinetics and Ergonomics, Rhodes University, Grahamstown, 6140 South Africa; 2 Department of Physiotherapy, College of Medicine, University of Ibadan, Ibadan, Oyo State Nigeria

**Keywords:** Exercise therapy, Muscle strength, Quadriceps muscle, Knee osteoarthritis

## Abstract

**Background:**

The study was designed to evaluate and compare the effectiveness of 12-week open, closed and combined kinetic-chain exercises (OKCE, CKCE and CCE) on quadriceps muscle strength and thigh girth of patients with knee osteoarthritis (OA).

**Method:**

The randomized clinical trial involved ninety-six consecutive patients with knee OA who were randomly assigned to one of OKCE, CKCE or CCE groups. Participants’ static quadriceps muscle strength (SQS), dynamic quadriceps muscle strength (DQS) and thigh girth (TG) were assessed using cable tensiometer, one repetition method and inelastic tape measure respectively at baseline and at the end of weeks 4, 8 and 12 of study.

**Results:**

The three groups were comparable regarding their demographic and dependent variables at baseline; there was significant time effect (*p* < 0.001each) as all three measures significantly increased over time from baseline to week 12 [mean difference: SQS: 3.30 (95% CI: 2.52–4.08) N; DQS: 0.74 (95% CI: 0.45–1.02) N; TG: 1.32 (95% CI: 0.93–1.71) cm]. The effect of intervention-time interaction was not significant (*p* > 0.05) for all three measures. Changes in SQS, DQS and TG between baseline and week 12 were also not significantly different (*p* > 0.05) among the three groups.

**Conclusion:**

All three exercise regimens are effective and demonstrate similar effects on quadriceps muscle strength and muscular trophism.

**Trial registration:**

NHREC/05/01/2008a. Registered 20th March, 2014 Retrospectively.

## Background

Osteoarthritis (OA) has become a major public health challenge because it causes chronic pain, reduces physical function and quality of life [1] and has been linked with increased ageing population and global prevalence of obesity [2]. The disease imposes a significant healthcare burden and accounts for high annual hospitalizations in the developed world [1, 3]. Chronic OA of the lower limb joints may lead to reduced physical fitness with a resultant increased risk of cardio metabolic co-morbidity [4, 5] and early mortality [6]. The disease which is characterized by complex multifactorial joint pathology is the most common form of joint disorder globally and majorly affects the knee [3, 7, 8].

The clinical features of chronic knee OA include pain, oedema and joint laxity which may lead to postural deformation [9, 10]. Long-term postural deformation causes the muscles to become fixed and rigid, resulting in reduced flexibility and abnormal gait [10]. Chronic knee OA leads to marked weakening of the quadriceps femoris muscle which is a major extensor and stabilizer of the knee [11]. Ageing and atrophy of this muscle result in knee pain and functional impairment [10]. International guidelines and findings from systematic reviews provide strong evidence in support of exercise therapy as first line non-pharmacological interventions for amelioration of symptoms [1, 12]. Fransen et al. [13] in a recent systematic review concluded that land-based therapeutic exercises generally provides benefits in terms of reduced knee pain and improved physical function and quality of life among people with knee OA. This suggests that therapeutic exercises meticulously planned to strengthen quadriceps muscle can relieve pain and lead to functional recovery of the muscle [14].

Exercises used for treatment of knee complaints are performed either in open or closed kinetic chain [15]. Open and closed kinetic chain exercises (OKCE and CKCE) have been shown to be individually effective for the improvement of quadriceps muscle strength in knee OA [16–18] but it appears there is no consensus regarding the comparative effectiveness of the two modes of exercise. A randomized controlled trial by Cho et al. [10] showed that CKCE improved electromyographic activities of all components of the quadriceps femoris muscle whereas OKCE did not show significant effect on vastus lateralis. However, the specific effects of CCE on muscle strength in the rehabilitation of knee OA has not been reported in literature. We conducted a randomized clinical trial to compare the effects of OKCE, CKCE and combined open and closed kinetic chain exercises (CCE) in patients with knee OA, founding that the latter produced significantly greater pain reduction than either OKCE or CKCE alone [19]. In the present study, we further analyzed the results of this trial on different outcome measures, i.e. on static and dynamic quadriceps strength and on muscular trophism.

## Methods

The study was approved by the Health Research Ethics Committee of the University of Ibadan and University College Hospital (Registration No: NHREC/05/01/2008a), the permission of the management of the Federal Medical Centre (FMC), Owo, Nigeria, was also obtained. All participants gave their informed consent before being included in the study. The participants were patients with mild to moderate knee OA (primary and secondary) attending the Physiotherapy Department, FMC, Owo between January 2013 and December 2014; they have been diagnosed according to the radiographic assessment of their knee joints by the orthopaedic surgeons and family physicians. They were male and females with knee OA of one or both knees with grade II Kellgren and Lawrence classification system based on plain x-rays taken in supine lying position [20]. They also satisfied the American College of Rheumatology Criteria for clinical classification of knee OA which were pain in the knee for most days of prior month, crepitation on active joint motion, morning stiffness less than 30 min in duration, patient’s age 38 years and above, and bony enlargement of the knee on examination [21]. The participants were also placed on 3000 mg Paracetamol daily. Potential participants who also had co-morbid neurological and severe systemic diseases as well as physical limitations that undermined their ability to cope with the demands of the study were excluded from the study.

The referring physicians/surgeons and the participants were blinded to participants’ assignment into the interventional groups but the researchers/assessors were not. Computer-generated random numbers were used to assign participants to one of Open Kinetic Chain Exercise (OKCE), Closed Kinetic Chain Exercise (CKCE) and Combined Chain Exercise (CCE) groups. Concealed codes (determined by the random numbers) were put on blank folders numbered 1–120 which were used by the physicians/surgeons to refer patients with knee OA to the researchers/assessors. A minimum sample size of 78 (26 per group) was determined using the Cohen’s table [22].

### Intervention

**Fig. 1:**
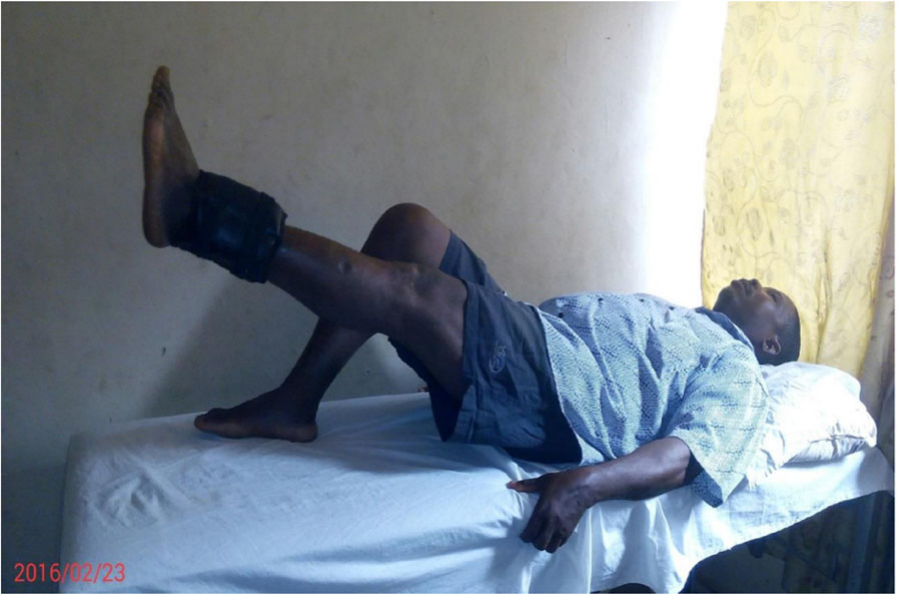
Participant performing (OKCE) straight leg raising with weight

**Fig. 2:**
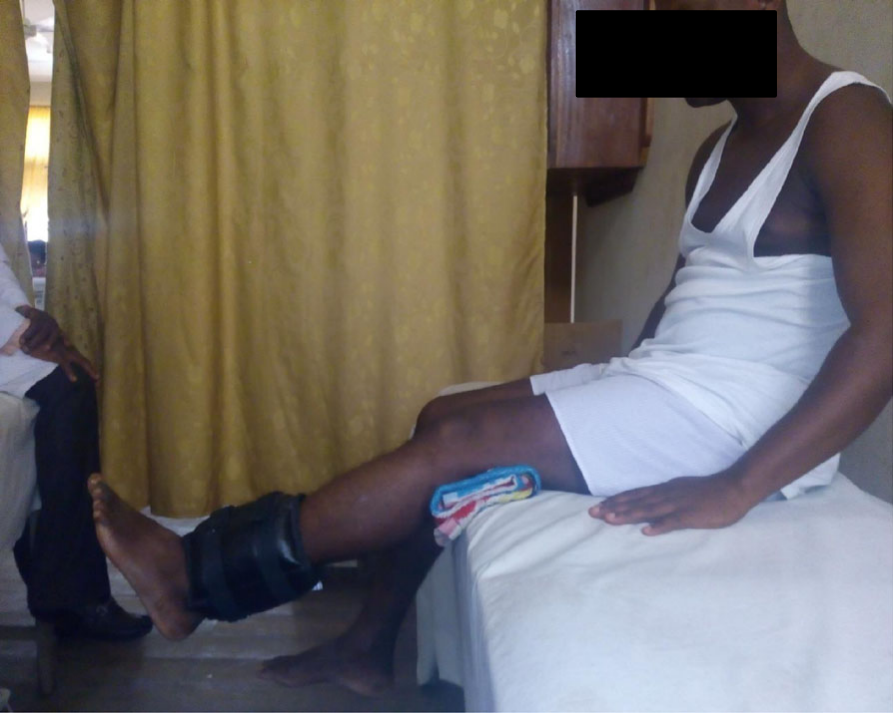
Participant performing (OKCE) full-arc extension exercise

**Fig. 3:**
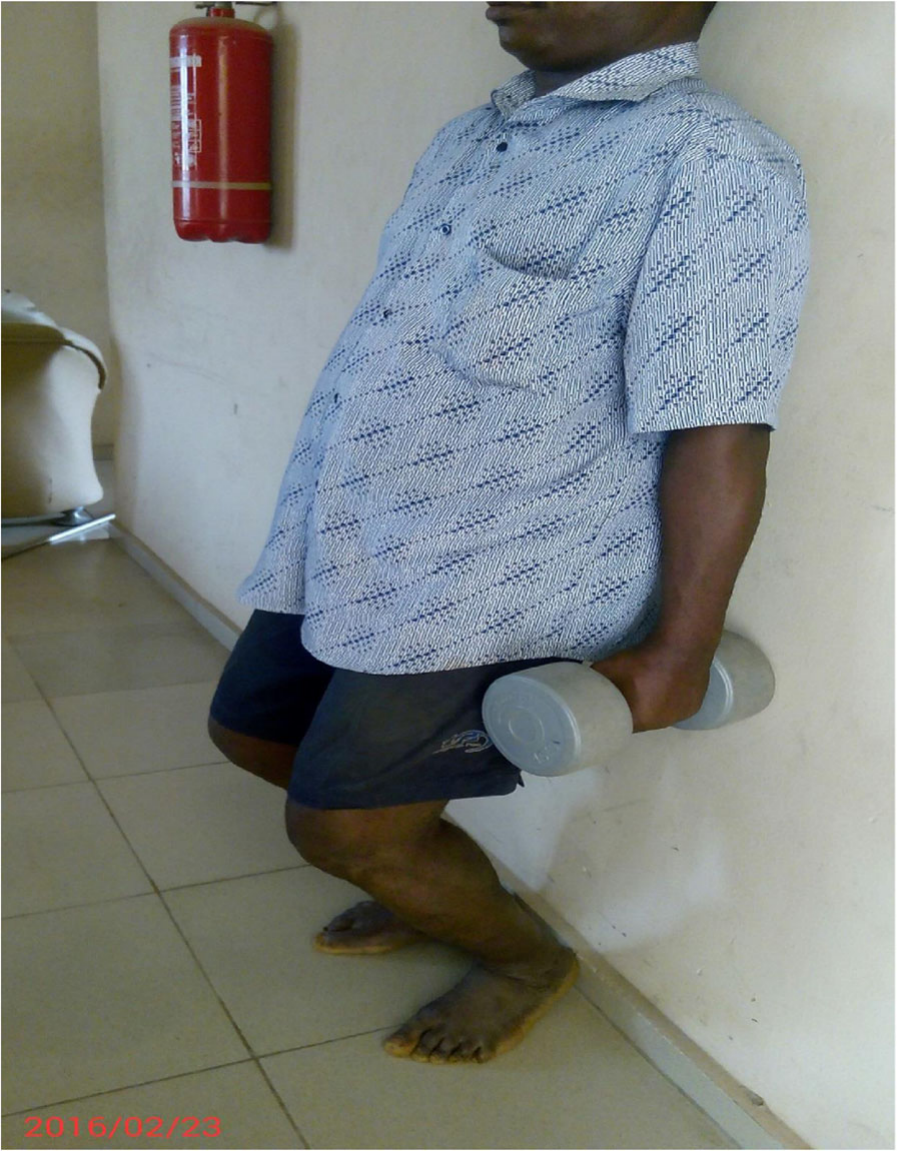
Participant performing (CKCE) wall slides with weight

**Fig. 4:**
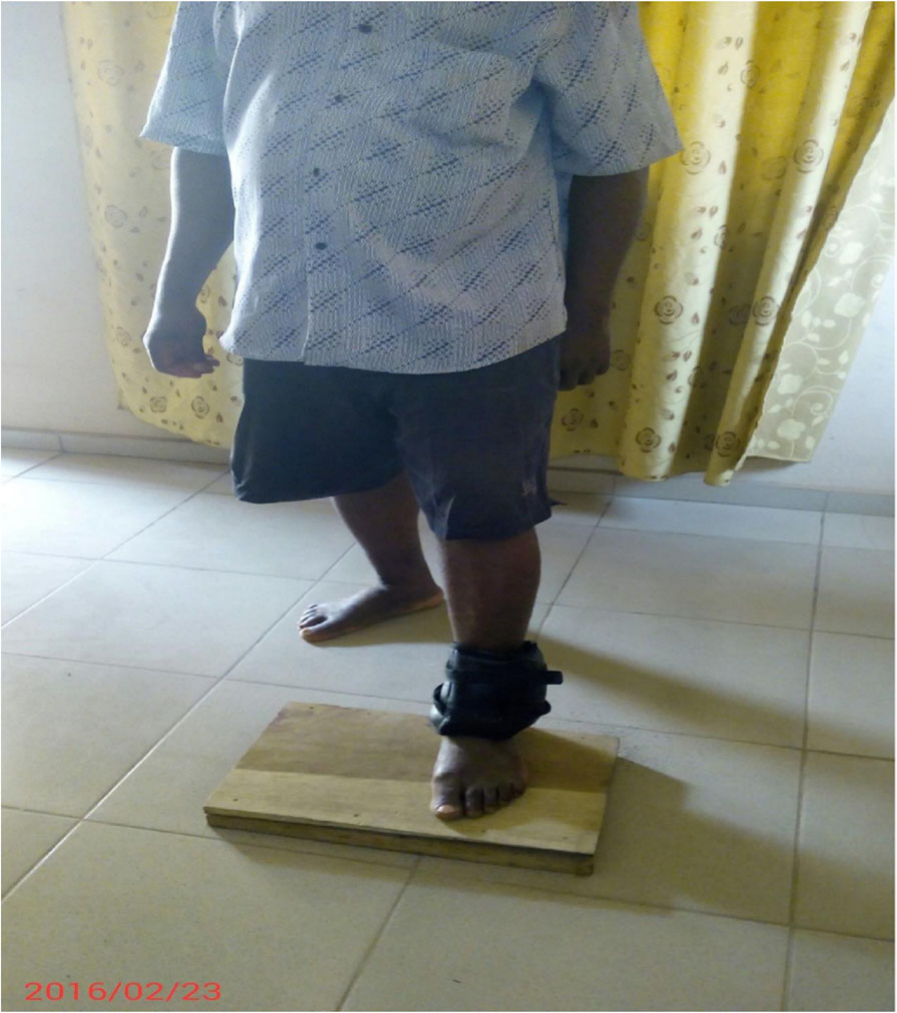
Participant performing (CKCE) steps up and down with weight

The exercise intervention protocol is described in details in the previous article [19] and is summarized in Table 1. Some of the intervention exercise protocols are illustrated in Figs. 1, 2, 3, 4.

**Table 1:** Summary of exercise training and progression for participants in OKCE, CKCE and CCE groups

Group/week	OKCE	CKCE	CCE
Week 1	(a) Quadriceps Setting (10 repetitions) (b) Cycling in the air (2 min for a bout)	(a) Quadriceps setting (10 repetitions) (b) Wall slides (10 repetitions)	(a) Straight leg raising (10 repetitions) (b) CKC Quadriceps setting (10 repetitions)
Week 2	(a) Quadriceps Setting (10 repetitions) (b) Cycling in the air (2 min for a bout) (c) Straight leg raising (10 repetitions)	(a) Quadriceps setting (10 repetitions) (b) Wall slides (10 repetitions)	(a) Straight leg raising (10 repetitions) (b) CKC Quadriceps setting (10 repetitions) (c) Wall slides (10 repetitions)
Week 3	(a) Quadriceps Setting (10 repetitions) (b) Cycling in the air (2 min for a bout) (c) Straight leg raising with weight (new 10 RM)	(a) Quadriceps setting (10 repetitions) (b) Wall slides with weight (new 10 RM)	(a) Straight leg raising with weight (new 10 RM) (b) CKC Quadriceps setting (10 repetitions) (c) Wall slides with weight (new 10 RM)
Week 4	(a) Quadriceps Setting (10 repetitions) (b) Cycling in the air (2 min for a bout) (c) Straight leg raising with weight (new 10 RM) (d) Full arc extension (with new 10 RM as weight)	(a) Quadriceps setting (10 repetitions) (b) Wall slides with weight (new 10 RM)	(a) Straight leg raising with weight (10 RM) (b) CKC Quadriceps setting (10 repetitions) (c) Wall slides with weight (new 10 RM) (d) Full arc extension (with new 10 RM as weight)
Week 5	(a) Quadriceps Setting (10 repetitions) (b) Cycling in the air (2 min for a bout) (c) Straight leg raising with weight (new 10 RM) (d) Full arc extension (with new 10 RM as weight)	(a) Quadriceps setting (10 repetitions) (b) Wall slides with weight (new 10 RM)	(a) Straight leg raising with weight (10 RM) (b) CKC Quadriceps setting (10 repetitions) (c) Wall slides with weight (new 10 RM) (d) Full arc extension (with new 10 RM as weight)
Week 6	(a) Quadriceps Setting (10 repetitions) (b) Cycling in the air (2 min for a bout) (c) Straight leg raising with weight (new 10 RM) (d) Full arc extension (with new 10 RM as weight)	(a) Quadriceps setting (10 repetitions) (b) Wall slides with weight (new 10 RM (c) Step up and down	(a) Straight leg raising with weight (10 RM) (b) CKC Quadriceps setting (10 repetitions) (c) Wall slides with weight (new 10 RM) (d) Full arc extension (with new 10 RM as weight)
Weeks 7–12	(a) Quadriceps Setting (10 repetitions) (b) Cycling in the air (2 min for a bout) (c) Straight leg raising with weight (new 10 RM) (d) Full arc extension (with new 10 RM as weight)	(a) Quadriceps setting (10 repetitions) (b) Wall slides with weight (new 10 RM (c) Step up and down with weight (new 10 RM)	(a) Straight leg raising with weight (10 RM) (b) CKC Quadriceps setting (10 repetitions) (c) Wall slides with weight (new 10 RM) (d) Full arc extension (with new 10 RM as weight)

10 repetitions of each exercise were carried out per session (except for full-arc extension and air cycling)

Cycling in the air (OKCE) was done continuously for 2 min for one bout of exercise

Three bouts of 10 repetitions of full-arc extension (OKC) were performed

Participants started with a weight equivalent to their 10RM and progressed by determining a new 10RM at the beginning of each week

Dumbells were used for wall slides

### Outcome measures

Static quadriceps muscle strength was assessed using cable tensiometer (Baseline, USA). Cable tensiometer has been reported to be reliable for measurements of static force of all muscle groups [15, 23]. Assessment of dynamic quadriceps muscle strength was done by predicting participants’ one repetition maximum (1-RM) through Brzycki equation [24]. The equation was reported to be valid for predicting 1-RM of lower limb muscles’ strength in adults and demonstrated high correlation with conventional 1-RM test (*r* = 0.92; *P* < 0.05) [25]. Thigh girth was assessed with tape measure (Butterfly, China). Lower extremity girth measurements has shown to be highly reliable, both intra and inter-tester (ICCs ranging from 0.82–1.0 and 0.72–0.97 respectively) [26].

### Assessment of muscle strength and thigh girth

#### Static muscle strength

**Fig. 5:**
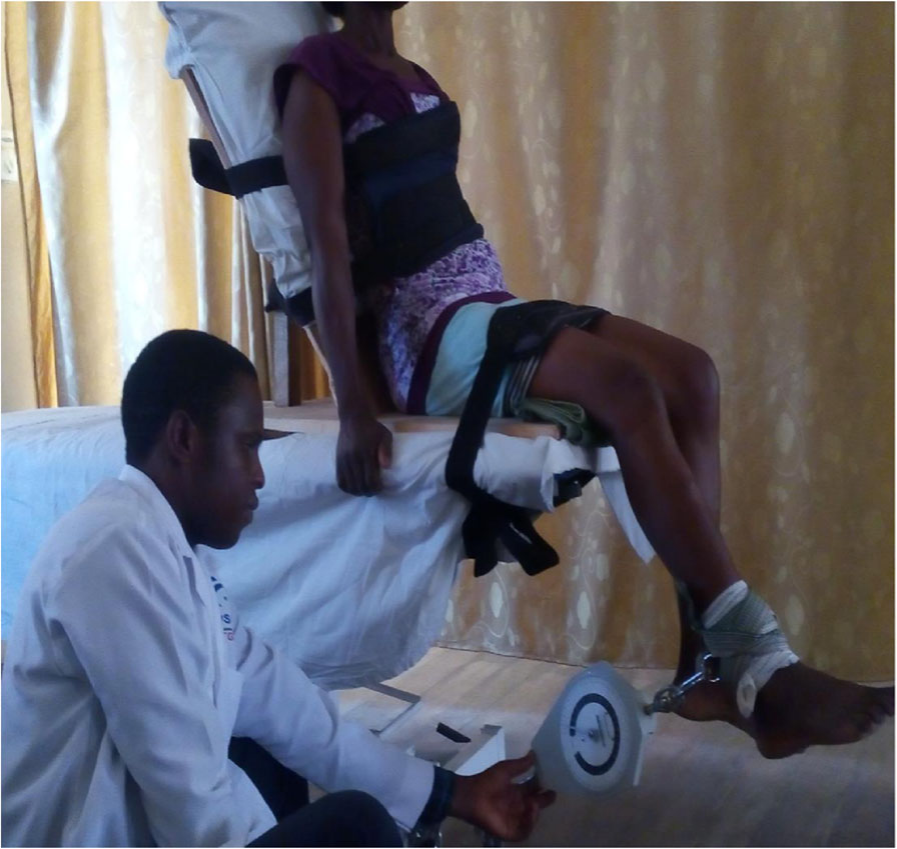
Participant during static quadriceps muscle strength testing

The participant sat on a testing table with his back resting firmly on the back rest, and the knees flexed over the edge of the testing table. The cable tensiometer was attached to the padded ankle of participant’s leg via its cable with the knee angle at 60 degrees of flexion (Adegoke BOA: Comparative efficacy of open and closed kinetic chain exercises in the treatment of osteoarthritic knee. PhD Thesis. Department of Physiotherapy, University of Ibadan; 2003, unpublished, [27]). After three trial tests, participants were instructed to hold the side of the testing table, look straight ahead, and try to straighten their knee with maximum effort without jerking (Adegoke BOA: Comparative efficacy of open and closed kinetic chain exercises in the treatment of osteoarthritic knee. PhD Thesis. Department of Physiotherapy, University of Ibadan; 2003, unpublished, [27]) (Fig. 5). After a rest of 90 s, the test was repeated and the average of the two attempts was taken and recorded in kilogrammes. Conversion to values in Newton (N) was done by multiplying the force measured in kilogrammes with acceleration due to gravity (9.8 m/s^2^). The converted static strength was normalised to body weight using the formula:

Sn = S/BW ^0.67^ [28].

S - Strength in Newton; BW – Body weight; 0.67 – allometric parameter.

The value obtained was recorded to nearest whole number as the participant’s static quadriceps muscle strength (SQS).

#### Dynamic quadriceps muscle strength

**Fig. 6:**
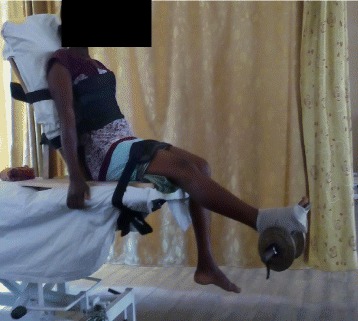
Participant during dynamic quadriceps muscle strength testing

Participants assumed a sitting position on a testing table with their back, and thighs well supported and firmly strapped to the backrest and sitting platform of the testing table respectively. A plastic weight (W) corresponding to a certain repetitive maximum of the participant was randomly selected and was attached via the De Lorme’s boot to the participant’s foot. Participants were instructed to lift the weight by extending their knee through the available range of motion. The participant held the position of maximum extension to a count of five and then returned to the starting position (Fig. 6). The lifting and lowering was terminated when the participant showed evidence of fatigue by not being able to complete the initial maximum range of motion [29]. The number of times the weight was lifted through full range of motion before fatigue set in was recorded as R. 1-RM was estimated using the formula predicted by: 1-RM = W/(1.0278–0.0278 x R) (kg) [24].

The value of 1-RM obtained in kilogrammes was converted to Newton and also normalized to body weight using the same procedure outlined for SQS [28] and was recorded as participant’s dynamic quadriceps muscle strength (DQS).

#### Thigh girth

The thigh girth (TG) was measured with an inelastic tape using a point mid-way along the straight line linking the landmarks of greater trochanter and the apex of the patella [30].

SQS, DQS and TG were assessed at baseline and at the end of weeks 4, 8 and 12 of study. For participants with bilateral knee OA, the index knees (with higher scores on the Visual Analogue Scale) were chosen for measurement [31].

### Data analysis

The data were analyzed using SPSS 20.0 version software (SPSS Inc., Chicago, Illinois, USA). Based on observations from a similar previous study [7], effect size of 0.8 and power of 80% were used to estimate a minimum sample size of 78 from the Cohen’s table [22]. Descriptive statistics of mean, standard deviation and percentages were used to summarize the data. Baseline variables were analyzed using one-way analysis of variance (ANOVA). The outcomes were analyzed according to the intention-to-treat principle. Data for all 96 participants were included in the analysis by carrying the last available score forward. We used a mixed model (repeated measures) ANOVA with intervention (OKCE, CKCE and CCE) as the between-subject variable and time (baseline, week 4, week 8 and week 12) as the within-subject variable. The dependent variables analyzed were SQS, DQS and TG. When significant intragroup differences were detected by the ANOVA, Bonferroni post-hoc was used to assess differences across baseline and weeks 4, 8 and 12.

Levels of significance was set at *p* = 0.05.

## Results

Ninety-six participants were included in the study. Thirty-two participants were randomly assigned to each of OKCE (14 male, 18 female), CKCE (12 male, 20 female) and CCE (11 male, 21 female) groups. Thirty-five of the participants (11OKCE, 12 CKCE, 12 CCE) presented with bilateral knee OA. Table 2 displays the demographic characteristics and clinical parameters of the groups; the 3 groups did not differ significantly at baseline regarding demographic characteristics and clinical parameters. The descriptive summary of SQS, DQS and TG at the four points of the study is also presented in Table 3.

**Table 2:** Comparison of participants’ demographic variables

	Groups				
	OKCE	CKCE	CCE		
	(*n* = 32)	(*n* = 32)	(*n* = 32)		
Variable	Mean ± SD	Mean ± SD	Mean ± SD	F-value	*p*-value
Age (Years)	63.50 ± 13.83	61.53 ± 12.94	58.78 ± 14.41	0.952	0.390
Height (m)	1.61 ± 0.07	1.60 ± 0.07	1.62 ± 0.06	1.466	0.465
Weight (kg)	79.34 ± 21.54	77.16 ± 18.08	77.13 ± 14.42	0.156	0.856
BMI (kg/m^2^)	31.22 ± 9.35	30.47 ± 8.32	29.46 ± 6.60	0.375	0.689

(*P* < 0.05)

*Key: OKCE* Open Kinetic Chain Exercise, *CKCE* Closed Kinetic Chain Exercise, *CCE* Combined Kinetic-Chain Exercises, *BMI* Body mass index

**Table 3:** Descriptive summary of static and dynamic quadriceps muscle strength and thigh girth at the four time-point of study

		Groups		
		OKCE (*n* = 32)	CKCE (*n* = 32)	CCE (*n* = 32)
Variable	Time	Mean ± SD (95% CI)	Mean ± SD (95% CI)	Mean ± SD (95% CI)
SQS (N)	Wk 0	7.02 ± 2.67 (6.05–7.98)	7.26 ± 2.60 (6.33–8.20)	7.46 ± 2.39 (6.59–8.32)
	Wk4	8.03 ± 2.65 (7.07–8.99)	8.24 ± 2.50 (7.34–9.14)	9.46 ± 2.73 (8.48–10.44)
	Wk8	9.03 ± 2.69 (8.06–10.00)	9.25 ± 2.45 (8.36–10.13)	10.42 ± 2.70 (9.44–11.39)
	Wk12	9.97 ± 2.78 (8.97–10.98)	10.25 ± 3.05 (9.15–11.35)	11.41 ± 2.67 (10.45–12.37)
DQS (N)	Wk 0	4.08 ± 0.68 (3.83–4.32)	4.28 ± 0.79 (4.00–4.57)	4.28 ± 0.65 (4.05–4.52)
	Wk 4	4.30 ± 0.69 (4.05–4.55)	4.50 ± 0.79 (4.22–4.78)	4.59 ± 0.77 (4.32–4.88)
	Wk 8	4.56 ± 0.70 (4.31–4.81)	4.75 ± 0.79 (4.46–5.03)	4.74 ± 0.80 (4.46–5.03)
	Wk12	4.75 ± 0.88 (4.44–5.07)	5.00 ± 1.10 (4.60–5.40)	5.11 ± 1.16 (4.69–5.53)
TG (cm)	Wk0	54.90 ± 7.19 (53.31–57.50)	53.19 ± 7.43 (50.51–55.90)	53.63 ± 5.69 (51.57–55.67)
	Wk4	55.06 ± 7.13 (52.49–57.63)	53.50 ± 7.33 (50.86–56.14)	54.28 ± 5.62 (52.25–56.31)
	Wk8	55.50 ± 7.04 (52.96–58.04)	54.09 ± 7.33 (51.45–56.74)	55.25 ± 5.55 (53.25–57.25)
	Wk12	55.91 ± 7.01(53.38–58.43)	54.47 ± 7.30 (51.84–57.10)	55.31 ± 5.70 (53.26–57.37)

*SD* Standard deviation, *Wk* week, *TG* Thigh girth, *OKCE* Open Kinetic Chain Exercise, *CKCE* Closed Kinetic Chain Exercise, *CCE* Combined Kinetic-Chain Exercises, *SQS* Static quadriceps muscle strength, *DQS* Dynamic quadriceps muscle strength, *TG* Thigh girth

**Fig. 7:**
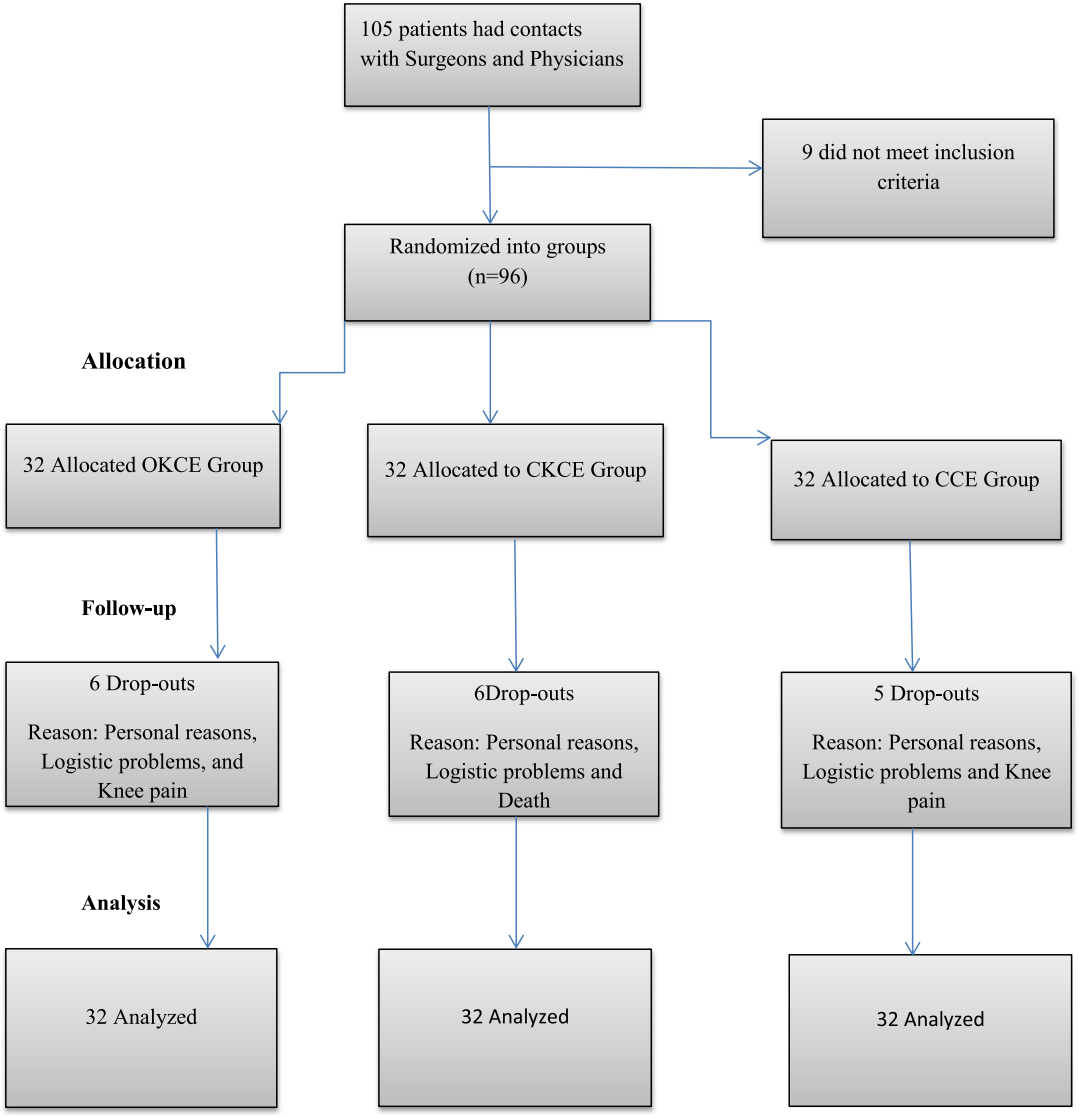
Flowchart of participants’ recruitment and participation

A total of 79 participants (26 OKCE, 26 CKCE, 27 CCE) concluded the protocol. Among the 13 participants who discontinued the protocol before the end of the fourth week, 7 of them (3 OKCE, 3 CKCE, 2 CCE) did not give salient reasons when contacted through telephone. The other 5 dropouts (2 OKCE, 2 CKCE, 1 CCE) were lost due to logistic problems such as inflexibility of time and venue of research being far from their places of abode. Among the 4 participants who discontinued the protocol during the eighth week of training, 3 (1 OKCE, 2 CCE) were lost to knee pain which became unbearable with exercise and the remaining one participant (CKCE) was lost to unexpected death from peptic ulcer complications. The flowchart of participants’ recruitment and participation in the protocol is presented in Fig. 7.

The analysis of time and intervention-time interaction effects on the variables is presented in Table 4. There was significant time effect (*p* < 0.001each) as all three measures significantly increased over time from baseline to week 12 [mean difference: SQS: 3.30 (95% CI: 2.52–4.08) N; DQS: 0.74 (95% CI: 0.45–1.02) N; TG: 1.32 (95% CI: 0.93–1.71) cm], with large effect sizes (SQS: 0.469; DQS: 0.274; TG: 0.426). There were significant increases (*p* < 0.05) for SQS, DQS and TG at all-time points of intervention. The effect of intervention-time interaction was not significant for all three measures [SQS (*p* = 0.347); DQS (0.834); TG (*p* = 0.984)].

**Table 4:** Analysis of time and interventionxtime interaction effects on static and dynamic quadriceps muscle strength and thigh girth

Variable	F		*p*		Partial Eta Square	
SQS (N)						
Time	82.015		<0.001		0.469	
Intvn*time	1.115		0.347		0.023	
DQS(N)						
Time	35.113		<0.001		0.274	
Intvn*time	0.225		0.834		0.005	
TG (cm)						
Time	1.864		<0.001		0.426	
Intvn*time	0.174		0.984		0.004	
Bonferroni post-hoc analysis of time effects						
	SQS (N)		DQS (N)		TG (cm)	
Time point (weeks)	Mean Difference (95% CI)	*P*	Mean Difference (95% CI)	*p*	Mean Difference (95% CI)	*P*
0 vs 4	1.33 (0.93–1.73)	<0.001	0.25 (0.18–0.32)	<0.001	0.38 (0.21–0.54)	<0.001
0 vs 8	2.32 (1.87–2.77)	<0.001	0.47 (0.38–0.56)	<0.001	1.04 (0.71–1.37)	<0.001
0 vs 12	3.30 (2.52–4.08)	<0.001	0.74 (0.45–1.02)	<0.001	1.32 (0.93–1.72)	<0.001
4 vs 8	0.99(0.82–1.16)	<0.001	0.22 (0.17–0.27)	<0.001	0.67 (0.46–0.87)	<0.001
4 vs 12	1.97 (1.21–2.73)	<0.001	0.49 (0.21–0.77)	<0.001	0.95 (0.67–1.23)	<0.001
8 vs 12	0.98 (0.25–1.71)	0.003	0.27 (0.00–0.54)	0.045	0.28 (0.06–0.51)	0.007

*p* < 0.05

*Vs* Versus, *Intvn* Intervention, *SQS* Static quadriceps muscle strength, *DQS* Dynamic quadriceps muscle strength, *TG* Thigh girth

Between group comparisons presented in Table 5 did not reveal significant difference among the three groups on any of the measures (SQS: *p* = 0.106; DQS: *p* = 0.291; TG: *p* = 0.660).

**Table 5:** Across group comparison of static and dynamic quadriceps muscle strength and thigh girth

Variable	F	*P*	Partial Eta Square
SQS (N)	2.296	0.106	0.047
DQS (N)	1.250	0.291	0.026
TG (cm)	0.417	0.660	0.009

*p* < 0.05

*SQS* Static quadriceps muscle strength, *DQS* Dynamic quadriceps muscle strength, *TG* Thigh girth

## Discussion

### Effects of open, closed and combined chain exercises on quadriceps muscle strength and thigh girth in knee osteoarthritis

The three exercise regimens produced significant improvements in static and dynamic quadriceps muscle strength which were also observed from the fourth week of study in the three intervention groups. The findings are in agreement with the reports of Anwer and Alghadir [32] who found significant increase in isometric quadriceps muscle strength for patients with knee OA after 5 weeks of isometric strength training programme. The results are also similar to the reports of previous related clinical trials [33, 34]. Durmus et al. [33] found a significant effect of a four-week biofeedback assisted isometric exercises on dynamic quadriceps muscle strength (1 and 10 RM) in their comparison of the exercise with electrical stimulation. Jan et al. [34] also reported a significant improvement in knee extensor peak torque in patients with OA who had 8-week weight bearing and non-weight bearing exercises. It was opined that quadriceps muscle weakness may be a primary risk factor for the development and progression of knee OA since weakness has been found to be present in very early joint degeneration [35]. Muscle strength declines are thought to primarily result from the atrophy of type IIB fibers, which are responsible for the rapid production of power [36]. Type IIB fibers have demonstrated the ability to hypertrophy after undergoing high tension and fatigue-inducing exercises [37] hence, muscle weakness is correctable with appropriate strength training programme [38]. A systematic review by Lange et al. [12] submitted that resistance training for patients with knee OA improved muscle strength in over 50–75% of cohort studies reviewed. Also, a summary of systematic reviews by Taylor et al. [39] on the positive and negative effects of progressive resistance exercises (PREs) identified increase in force generating capacity of the muscles as a benefit of PREs in patients with osteoarthritis and other musculoskeletal conditions. The OKCE and CKCE adopted by the present study are progressive resistance exercises which have also been described as valid method of increasing the ability of the muscles to generate force [15] hence the observed significant increase in quadriceps muscle strength.

Participants in OKCE, CKCE and CCE groups showed significant improvements in thigh girth at weeks 8 and 12 of the study. It has been reported that the initial rapid gain in the tension-generating capacity of skeletal muscle in resistance training is largely attributed to neural responses and not adaptive changes in muscle itself [40, 41]. Hypertrophy is an increase in the size (bulk) of an individual muscle fiber caused by an increase in myofibrillar volume [42, 43]. Following a moderate to high-intensity resistance training of about 4 – 8 weeks [15] or 2 – 3 weeks of very high-intensity resistance training [44] hypertrophy becomes an increasingly important adaptation that accounts for strength gains in muscle [15]; the significant increase in thigh girth demonstrated by the three groups in this study may be evidence in supports these viewpoints, although there is an opinion in literature that unnoticeable increase in fat deposits in the thigh could cause increase in thigh girth during thigh muscles’ strength training especially when the study population is dominated by the female [23]. However, Adegoke (Adegoke BOA: Comparative efficacy of open and closed kinetic chain exercises in the treatment of osteoarthritic knee. PhD Thesis. Department of Physiotherapy, University of Ibadan; 2003, unpublished) and Miyaguchi et al. [29] did not observe significant effects of quadriceps strengthening exercises on the thigh girth of individuals with knee OA. Adegoke (Adegoke BOA: Comparative efficacy of open and closed kinetic chain exercises in the treatment of osteoarthritic knee. PhD Thesis. Department of Physiotherapy, University of Ibadan; 2003, unpublished) attributed his finding to the fact that the majority of the studied participants were women who have been reported not to develop hypertrophy like men, and the probability that the intensity of exercise used was not tasking enough. The exercise intensity used in this study is similar to Adegoke’s protocol but for a longer duration (8 weeks versus 12 weeks) and some few methodical differences in terms of sample size and test statistics. Miyaguchi et al. [29] did not find any significant effect of 12-week static exercise on the thigh circumference of their participants. The present study incorporated both static and dynamic exercises in each of OKCE, CKCE, and CCE which could have enhanced faster hypertrophy of participant’s thigh muscles.

### Comparative effectiveness of open, closed and combined chain exercises on quadriceps muscle strength and thigh girth

Juhl et al. [45] in a systematic review and meta-analysis of randomized controlled trials identified quadriceps strengthening as one of the major aim and focus of optimal exercise programme for patients with knee OA; Tinaka et al. [46] in their meta-analysis also affirmed that muscle strengthening exercises (with or without weight bearing and aerobic exercises) are effective for pain relief; hence it is relevant to identify the best approach to improving quadriceps muscle strength for optimal clinical benefits.

The results of this study suggest that the three modes of kinetic-chain exercises compared had similar effects on SQS, DQS and TG. Alghamdi et al. [47] in a review of literature suggested the use of CCE arguing that clinicians should not rule out CKCE in the management of knee OA because of the concerns of their potentials to possibly induce wear and tear of joint cartilage which might accelerate disease progression. Open kinetic chain exercises are better for isolated quadriceps muscle strengthening while CKCE encourages co-contraction of other muscle groups with the body weight providing additional resistance [15, 48]. The superiority of CCE over either OKCE or CKCE in terms of pain reduction reported in our previous article [19] was attributed to the combination of aforementioned features in CCE. The force generating capacity of skeletal muscles of individuals with similar population characteristics are likely to have comparable improvements if subjected to similar overloads [15]. The three modes of kinetic-chain exercises employed in this study are comparable in intensity and progression.

Studies on the effectiveness of CCE in knee OA are rather scarce The effectiveness of combining OKCE and CKCE in post anterior cruciate ligament (ACL) reconstruction [49] and patellofemoral pain syndrome (PPS) [50] (among younger population) have also been reported in literature. Mikkelsen et al. [49] found that addition of isokinetic OKCE to CKCE for one study group at the sixth week after both groups commenced CKCE produced significantly higher isokinetic quadriceps strength than the group that had CKCE alone, isokinetic strength training and assessment were however not considered in present study. Minoonejad and colleagues [50] did not assess muscle strength although they reported significantly more reduction in pain for participants with PPS who had CCEs than the controls who did not undergo any exercise training; hence the extent to which their findings can be compared with the results of this study is limited.

Unlike CCE, the effectiveness of OKCE and CKCE on quadriceps muscle strength in knee OA have been well documented in literature ([16–18, 34], (Adegoke BOA: Comparative efficacy of open and closed kinetic chain exercises in the treatment of osteoarthritic knee. PhD Thesis. Department of Physiotherapy, University of Ibadan; 2003, unpublished)); some of the authors [16–18] found CKCE to have produced significantly higher quadriceps muscle strength than OKCE while the two modes of exercises were reported to be equal in their effects on quadriceps muscle strength in some other studies (Adegoke BOA: Comparative efficacy of open and closed kinetic chain exercises in the treatment of osteoarthritic knee. PhD Thesis. Department of Physiotherapy, University of Ibadan; 2003, unpublished, [34]).

### Clinical implication of study

The study’s outcome indicated that OKCE, CKCE and CCE are all effective for improving static and dynamic muscle strength and thigh muscle bulk of patients with knee OA. Hence, the results suggest all three exercise regimens can be employed in isolation for improvement of quadriceps muscle function and performance in this category of patients. However the results should be interpreted with caution considering the proportion of drop-outs from the trial.

### Limitations of the study

Our study is not without limitations; the assessors were not blinded to participant’s interventional group assignment, although the researchers did their best to minimize assessment-related bias by ensuring that a neutral research assistant recorded all data into data spreadsheet. It is probable such bias might have introduced some confounding factors capable of threatening the internal validity of this study.

The effects of 3000 mg Paracetamol on the findings of this study were not evaluated; information on mean number of the medication taken by each participant on weekly/monthly basis could have been helpful in interpreting the results of this study. The large number of dropouts (17.7%) might have undermined the power of this study which in turn may have negatively impacted on the external validity of the study. Lack of a control group with knee OA undergoing sham/no intervention is another limitation of this study. This would have shown the real treatment effects by eliminating any placebo effects produced by the intervention groups.

## Conclusion/recommendation

The findings of this study have shown that OKCE, CKCE and CCE are all effective for improving quadriceps muscle strength in patients with knee OA and almost equal in their effects on the force-generating capacity of the muscle. Future studies should verify whether the three protocols have different effects when the treatment is conducted over a longer period. Information on the comparative effects of the three exercise regimens on other clinical and psychosocial variables in knee OA will also be good for future perspective. Designing a follow-up phase aimed at investigating the sustainability of observed improvements may also be considered.

## Abbreviations

1-RM: One repetitive maximum
CCE: Combined kinetic-chain exercise
CKCE: Closed kinetic-chain exercise
DQS: Dynamic quadriceps muscle strength
OKCE: Open kinetic-chain exercise
SQS: Statiic quadriceps muscle strength
TG: Thigh girth
